# Knowledge, attitude, and practice among nurses regarding the prevention of pressure ulcers in a tertiary care hospital: a cross-sectional study

**DOI:** 10.1038/s41598-025-18303-4

**Published:** 2025-10-23

**Authors:** Fawad Inayat, Hafiza Saba Javed, Summaya Inayat, Numan Khan, Junaid Shakir, Amna Khalid, Muhammad Jawad Ullah

**Affiliations:** 1 Department of Biochemistry, Abdul Wali Khan University, Mardan, Pakistan; 2 Department of Public Health, Chiang Mai University Mueang Chiang Mai District, Chiang Mai, 50200 Thailand; 3 Department of Chemistry, Shaheed Benazir Bhutto Women University, Peshawar, 25000 Pakistan; 4 Department of Oral and Maxillofacial Surgery, Lady Reading Hospital, Peshawar, Pakistan; 5 Department of Allied Health Sciences, Iqra National University, Peshawar, Pakistan; 6 Aziz Fatima Medical and Dental College, Faisalabad, Pakistan

**Keywords:** Pressure ulcers, Bedsores, Pressure injuries, Nurse knowledge, Nurse attitudes, Nurse practices, Healthcare, Prevention, Faisalabad, Pakistan, Health care, Health policy, Health services

## Abstract

This study aimed to evaluate nurses’ knowledge, attitudes, and practices (KAP) regarding pressure ulcer prevention in a tertiary care hospital. A cross-sectional design was employed to assess nurses’ KAP regarding pressure ulcer (PU) prevention. The study was conducted at the Government Hospital of Faisalabad, Pakistan. A purposive sampling method selected 200 registered nurses currently employed at the hospital. The average age was 30.20 ± 5.61 years, with 75% (*n* = 150) married and 92% (*n* = 182) holding a diploma in nursing. Knowledge regarding pressure ulcers was high, with 49.5% (*n* = 98) strongly agreeing and 31.8% (*n* = 61) agreeing that pressure ulcers cause severe illnesses, yielding a Likert score of 4.15. Awareness of the Braden Scale was also high, with 50% (*n* = 99) strongly agreeing and 31.3% (*n* = 62) agreeing, resulting in a score of 4.18. The practice of turning patients every two hours was well-received, with 47.5% (*n* = 94) strongly agreeing, leading to a score of 4.10. Age (*p* = .134), marital status (*p* = .571), and level of education (*p* = .072) were not significant predictors of knowledge scores. However, higher knowledge scores significantly predicted more positive attitudes (*p* < .001) and better practices (*p* < .001) in pressure ulcer prevention. The study highlights significant gaps in nurses’ knowledge, attitudes, and practices regarding PU prevention. The findings underscore the need for continuous education and training to enhance nurses’ competence in PU prevention. Addressing these gaps through targeted interventions can improve patient outcomes and reduce the prevalence of PUs in healthcare settings.

**Supplementary Information:**

The online version contains supplementary material available at 10.1038/s41598-025-18303-4.

## Introduction

Pressure ulcers, also known as bedsores or pressure injuries, are localized damage to the skin and underlying tissue typically over a bony prominence because of prolonged pressure or pressure combined with shear^1,2^. These injuries represent a significant concern in healthcare settings, particularly for immobile or bedridden patients^3,4^. The development of pressure ulcers can lead to severe complications, including infections, prolonged hospital stays, and increased morbidity and mortality^5^. The global prevalence of pressure ulcers in hospitalized patients varies, with studies indicating rates ranging from 8.3 to 25.1% in Europe, the United States, Canada, and Australia^6–8^.

The etiology of pressure ulcers is multifactorial, with primary causes including unrelieved pressure, shear forces, friction, and moisture^8^. Contributing factors to the development of pressure ulcers encompass a variety of patient-related and environmental factors. These include poor nutrition, reduced mobility, diminished sensory perception, incontinence, and dehydration. Additionally, advanced age and the presence of comorbid conditions such as diabetes, hypertension, and ischemic heart disease further elevate the risk of pressure ulcer formation^8,9^.

The role of nurses is crucial in preventing and managing pressure ulcers. Nurses are often the first healthcare professionals to detect early signs of pressure damage and are responsible for implementing preventive measures^10,11^. Effective prevention strategies involve regularly repositioning patients, using pressure-relieving devices, maintaining skin hygiene, and ensuring adequate nutrition and hydration. Despite the critical role of nurses in pressure ulcer prevention, research indicates varying levels of knowledge, attitudes, and practices (KAP) among nursing staff regarding this aspect of patient care^12,13^.

A positive attitude towards pressure ulcer prevention among nurses is essential for effectively implementing preventive measures. Attitude refers to the predisposition to respond positively or negatively to a particular idea, object, person, or situation. It encompasses the beliefs, feelings, and behavioral tendencies towards pressure ulcer prevention. Nurses’ attitudes are influenced by their knowledge and understanding of the importance of pressure ulcer prevention, as well as their confidence in their ability to implement preventive measures effectively^14,15^.

Despite the availability of guidelines and educational resources, nurses’ knowledge and practice gaps persist. Several studies have highlighted the need for ongoing education and training to enhance nurses’ competence in pressure ulcer prevention^16,17^. For instance, a study conducted by Nurhusien Nuru et al. (2015) in Ethiopia revealed inadequate knowledge and practice among nurses, with more experienced nurses and those with higher educational levels exhibiting better attitudes towards pressure ulcer prevention^18^. Similarly, research by Bayan Kaddoura et al. (2016) in Saudi Arabia found that healthcare professionals had low levels of knowledge and unsatisfactory attitudes towards pressure ulcer prevention, emphasizing the need for increased awareness and education^19^.

In addition to knowledge and attitudes, the practice of preventive measures is a critical component in reducing the incidence of pressure ulcers. Practical interventions such as regular patient repositioning, skin assessments, and the use of pressure-relieving devices are essential. Studies have shown that nurses with greater knowledge of pressure ulcer staging and prevention are more likely to engage in effective preventive practices^20^.

Despite the critical role of nurses and the availability of evidence-based guidelines, the prevalence of pressure ulcers remains high, particularly in developing countries with limited healthcare resources^21,22^. In Pakistan, data on the occurrence of pressure ulcers is scarce, highlighting the need for local studies to understand the extent of the problem and the effectiveness of preventive measures^23–25^. The financial burden of treating pressure ulcers is substantial, with estimates suggesting that treatment costs are 3–4 times higher than prevention costs^26,27^.

In Pakistan, the prevalence and management of pressure ulcers remain understudied, particularly in tertiary care settings like Faisalabad. Limited resources, high patient-to-nurse ratios, and gaps in staff training contribute to the challenges in effective PU prevention. Local studies are scarce, and existing data suggest that preventive practices may not be uniformly implemented. This study aims to fill this gap by assessing nurses’ KAP in a tertiary care hospital in Faisalabad, providing insights that can guide interventions to reduce PU incidence and improve patient outcomes in similar resource-constrained settings.

## Materials and methods

### Study design

This study employed a cross-sectional design to evaluate nurses’ knowledge, attitude, and practice (KAP) regarding preventing pressure ulcers in a hospital setting. Cross-sectional studies are well-suited for assessing the prevalence and relationships of various characteristics within a specific population at a given point in time.

### Study setting

The research was conducted at the Government Hospital, from January 1 st, 2021, to July 30th, 2021. This hospital serves a diverse patient population and employs many registered nurses, making it an appropriate setting for this study.

### Sample size and sampling technique

A non-probability purposive sampling method was used to select the study participants. The study aimed to include 200 registered nurses currently employed at the hospital. Purposive sampling was chosen to ensure that participants had relevant experience and exposure to patients at risk of pressure ulcers. This approach facilitated the collection of rich, pertinent data.

### Inclusion and exclusion criteria

#### Inclusion criteria

- Registered nurses currently working in the hospital.
- Nurses who provided informed consent to participate in the study.

#### Exclusion criteria

- Student nurses and midwives.
- Nurses who were unwilling to participate.

### Data collection tool

Data was collected using a structured, self-administered questionnaire developed specifically for this study. The questionnaire assessed the nurses’ knowledge, attitudes, and practices related to pressure ulcer prevention and management.

### Questionnaire structure

A self-structured was used in this study (Supplementary File 1). Age, gender, marital status, educational level, and years of nursing experience. Consisted of 12 multiple-choice questions focusing on understanding pressure ulcer etiology, risk factors, prevention strategies, and management practices. Included 7 statements rated on a 5-point Likert scale (strongly agree, agree, neutral, disagree, strongly disagree) to gauge the nurses’ attitudes towards pressure ulcer prevention and their perceived importance of various preventive measures. Comprised 6 multiple-choice questions evaluating the preventive practices nurses implement in their daily routines, such as patient repositioning frequency, pressure-relieving devices, and skin assessment protocols.

### Data collection procedure

Data collection was carried out over three months. The following steps were adhered to:

An initial orientation session was conducted to explain the purpose and procedure of the study to the potential participants. During this session, the importance of accurate and honest responses was emphasized. Questionnaires were distributed to the selected nurses during their shift breaks or after work hours to minimize disruption to their duties. Participants were given 15–20 min to complete the questionnaire in a quiet, designated area within the hospital. Completed questionnaires were collected in sealed envelopes to ensure the confidentiality of the responses. Participants were assured that the data would be used solely for research purposes and that their identities would not be disclosed.

### Data analysis

Data was entered and analyzed using Statistical Package for the Social Sciences (SPSS) software version 21. The following statistical techniques were employed: Frequencies and percentages were calculated for categorical variables. Means and standard deviations were computed for continuous variables. Chi-square tests assessed the association between demographic variables and knowledge, attitude, and practice scores. Multivariate regression analysis was conducted to determine knowledge, attitudes, and practices predictors.

## Results

### Demographics

The average age of the participants was 30.20 + 5.61, with a minimum age of 23 and a maximum age of 49. There were 38 nurses between the ages of 18 and 27, 62 nurses between the ages of 28 and 37, 74 nurses between the ages of 38 and 47, and 26 nurses over the age of 48. Most of the nurses (*n* = 150) were married; however, some were single (*n* = 38), widowed (*n* = 6), and divorced (*n* = 4). Most of the nurses (*n* = 182) had a diploma, and only a tiny percentage (*n* = 16) had a bachelor’s degree in nursing. None of the nurses had earned a master’s degree in nursing, as shown in Table 1.

**Table 1:** Demographic data of Participants.

Variables	Details	Frequency (%)
Age	(Mean *±* S.D)	30.20 *±* 5.61
Marital status	Single	38 (19%)
	Married	150 (75%)
	Divorced	4 (2%)
	Widowed	6 (3%)
Level of education	Diploma in Nursing	182 (92%)
	Bachelor of Nursing	16 (8%)
	Master of Nursing	0 (0)

### Knowledge

The responses indicate that nurses have a solid understanding of the critical aspects of pressure ulcer management. A significant majority, 98 (49.5%) strongly agreed and 61 (31.8%) agreed that pressure ulcers cause severe illnesses such as septic shock, respiratory failure, and acute renal failure, yielding a Likert score of 4.15. Furthermore, knowledge regarding the Braden Scale was evident, with 99 (50.0%) strongly agreeing and 62 (31.3%) agreeing on its use for assessing and grading pressure ulcers, resulting in a score of 4.18. The awareness that skin over bony prominences is more prone to developing pressure ulcers was high, with a score of 4.11, supported by 101 (51.0%) strongly agreeing. The understanding that chronic bedridden and wheelchair users are at higher risk was also substantial, with a score of 4.02 as shown in Table 2.

Knowledge of the role of obstructed blood flow to soft tissue in forming pressure ulcers was confirmed by a score of 4.08, with 101 (54.0%) strongly agreeing. Neuropathy and paralysis were recognized as factors reducing skin sensitivity and contributing to pressure ulcers, with 104 (52.5%) strongly agreeing, leading to a score of 4.01. The risk posed by skin wetness was acknowledged with a score of 3.94 as shown in Table 2. All Likert-scale responses were analyzed as continuous variables (range: 1–5), with mean scores > 4.0 indicating strong agreement (e.g., 4.15 ± 0.72).

### Attitude

Nurses exhibited positive attitudes toward various preventive measures and treatments for pressure ulcers. The importance of frequent repositioning of patients and maintaining wrinkle-free bed sheets was recognized, with 90 (45.5%) strongly agreeing, yielding a score of 4.09. Attitudes towards the use of hydra colloid dressing were moderately positive, with a score of 3.80 as shown in Table 2.

The utilization of saline water for cleaning wounds was well-received, with a score of 4.01. There was a recognition of the delaying effect of anti-inflammatory drugs on the healing process of pressure ulcers, with a score of 4.03. The role of ripple mattresses and cushions in prevention was debated, with the lowest score of 3.47, indicating divided opinions as shown in Table 2.

The necessity of proper skin care and adequate nutrition in preventing pressure ulcers was well-supported, scoring 4.04. The efficacy of wound care through debridement and dressing was also positively regarded, scoring 4.05. The importance of proper repositioning protocols was acknowledged, scoring 4.09. However, there was a notable debate on the role of massage over bony prominences, scoring 3.88, reflecting mixed attitudes as shown in Table 2.

### Practices

Nurses’ practices in pressure ulcer prevention and management were positively rated. The practice of turning patients every two hours to prevent bedsores scored highly, with 94 (47.5%) strongly agreeing, leading to a score of 4.10. Attention to pressure points during skin cleansing was also emphasized, scoring 4.01. The practice of assessing every patient for the development of pressure ulcers upon hospital admission was supported, with a score of 3.77 as shown in Table 2.

Educational programs aimed at reducing the development of pressure ulcers were viewed favorably, scoring 3.97. However, practices involving head elevation at 30 degrees were less positively rated, with a score of 3.48, reflecting a need for clearer guidelines or better understanding as shown in Table 2.

### Preventing pressure ulcers that nurses practice

The usual nursing care for bed ulcers was the subject of eleven questions. More than half of the nurses have good bedsore care and preventive experience, whereas the rest have little or no experience with pressure ulcer prevention and care. Massage on bony prominence areas is used by 87 (43.9%) of nurses to reduce the incidence of Pressure Ulcers, while 50 (25.3%) of nurses do not perform massage. 94 (47.5%) of nurses change their body position every two hours to reduce the risk of developing a pressure ulcer, while 30 (15.2%) of nurses do not follow this positioning protocol as shown in Table 2.

**Table 2:** The likert scale scoring for nurse’s knowledge, attitude, and practice.

Questions	Strongly agree *N* (%)	Agree on *N* (%)	Neutral *N* (%)	Disagree *N* (%)	Strongly Disagree *N* (%)	Likert Score
Pressure ulcer causes serious illnesses suchas septic shock, respiratory failure, acute renal failure, etc.	98 (49.5)	61(31.8)	10 (5.1)	29(14.6)	0 (0)	4.15
Braden Scale used for assessment and grading of pressure ulcers.	99 (50.0)	62 (31.3)	10 (5.1)	27 (13.6)	0 (0)	4.18
The skin over bony prominence is more prone to develop pressure ulcers.	101(51.0)	52(26.3)	11 (5.6)	34 (17.2)	0 (0)	4.11
Chronic bedridden and wheelchair users are more at risk of developing pressure ulcers.	93 (47.0)	57(28.8)	6 (3.0)	41 (21.2)	0 (0)	4.02
Completelyor partially obstructed blood flow to soft tissue leads to the formation of pressure ulcers	101 (54.0)	40(20.2)	11 (5.6)	40 (20.2)	0 (0)	4.08
Neuropathy and paralysis cause pressure ulcers by reducing the sensitivity of the skin.	104 (52.5)	43(21.7)	3 (1.5)	44 (22.2)	4 (2.0)	4.01
The risk of pressure ulcers is increased by skin wetness (e.g. urine incontinence, stool, sweating, etc.).	100 (50)	45(22.7)	8 (4.0)	31 (15.7)	14 (7.1)	3.94
2nd Stage of pressure ulcer is the involvement of muscle, bone, and connective tissue.	92 (46.5)	54(27.3)	9 (4.5)	34 (17.2)	9 (4.5)	3.94
Frequent repositioning of patients and wrinkle-free bed sheets prevent pressure ulcers.	90 (45.5)	67(33.8)	10 (5.1)	31 (15.7)	0 (0)	4.09
Hydra colloid dressing is used for patients with pressure ulcers.	74 (37.4)	67(33.8)	11 (5.6)	36 (18.2)	10 (5.1)	3.80
Saline water is used to clean the wounds of pressure ulcers.	80 (40.4)	69(34.8)	19 (9.6)	30 (15.2)	0 (0)	4.01
Anti-inflammatory drugs delay the healing process of pressureulcer wounds.	86 (43.4)	65(32.8)	14 (7.1)	33 (16.7)	0 (0)	4.03
Ripple mattresses and cushions have no role in the prevention of pressure ulcers.	71 (35.9)	48(24.2)	10 (5.1)	42 (21.2)	27(13.6)	3.47
Proper skin care and adequate nutrition prevent pressure ulcers.	97(49.0)	52(26.3)	9 (4.5)	40 (20.2)	0 (0)	4.04
Best wound care by debridement and dressing faster the healing process of pressure ulcer.	91 (46.0)	59(29.8)	15 (7.6)	33 (16.7)	0 (0)	4.05
Friction occurs when moving the patient up in bed	91 (46.0)	61(30.8)	11 (5.6)	35 (17.7)	0 (0)	4.05
Placement of pillow under patient’s leg help in the prevention of bedsore	94 (47.5)	54(27.3)	10 (5.1)	40 (20.2)	0 (0)	4.02
For proper repositioning protocol, a turning schedule should be written and placed on the bedside of patients.	98 (49.5)	56(28.3)	8 (4.0)	36 (18.2)	0 (0)	4.09
Massage over bony prominences help in the prevention of pressure ulcer	87 (43.9)	50(25.3)	11 (5.6)	50 (25.3)	0 (0)	3.88
Dragging the patient during repositioning doesn’t cause pressure ulcers.	76 (38.4)	58(29.3)	9 (4.5)	41(20.7)	14(7.1)	3.71
Turn the patient position every two hours to prevent bedsore	94(47.5)	60(30.3)	14 (7.1)	30 (15.2)	0 (0)	4.10
While cleansing of skin paying more attention to pressure points helps in the prevention of bedsore	94 (47.5)	51(25.8)	13 (6.6)	40 (20.2)	0 (0)	4.01
Every patient on admission to the hospital should be assessed for the development of pressure.	78 (39.4)	52(26.3)	12 (6.1)	56 (28.3)	0 (0)	3.77
Education programs may decrease the ratio of Development of pressure ulcers.	88 (44.4)	56(28.3)	14 (7.1)	40 (20.2)	0 (0)	3.97
Head elevation should be 30 degrees consistent with a medical condition.	70 (35.4)	52(26.3)	12 (6.1)	32 (16.2)	32 (16.2)	3.48

### Knowledge score regression

Age did not significantly predict knowledge scores about pressure ulcers (B = 0.01, SE = 0.01, t = 1.51, *p* =.134), with a confidence interval of −0.00 to 0.03. Marital status also was not a significant predictor (B = 0.05, SE = 0.09, t = 0.57, *p* =.571), with a confidence interval of −0.13 to 0.23. Level of education approached significance (B = −0.28, SE = 0.16, t = −1.82, *p* =.072); the negative coefficient suggests that nurses with higher education levels might have slightly lower knowledge scores, with a confidence interval of −0.59 to 0.03. The intercept for this model was 3.80 (SE = 0.39, t = 9.82, *p* <.001), indicating the baseline knowledge score when age, marital status, and level of education are held constant, as shown in Table 3.

### Attitude score regression

Age did not significantly predict attitude scores towards pressure ulcer prevention and management (B = 0.01, SE = 0.01, t = 0.84, *p* =.402), with a 95% confidence interval of −0.01 to 0.02. Marital status also was not significant (B = −0.08, SE = 0.08, t = −0.97, *p* =.333), with a confidence interval of −0.25 to 0.09. Level of education did not significantly predict attitude scores (B = 0.12, SE = 0.14, t = 0.83, *p* =.408), with a confidence interval of −0.17 to 0.40. The intercept for this model was 3.70 (SE = 0.36, t = 10.32, *p* <.001), indicating the baseline attitude score when age, marital status, and level of education are held constant. The knowledge score significantly predicted attitude scores (B = 0.67, SE = 0.07, t = 9.78, *p* <.001), with a 95% confidence interval of 0.53 to 0.81, indicating that higher knowledge scores are associated with more positive attitudes towards pressure ulcer prevention and management, as shown in Table 3.

### Practices score regression

Age did not significantly predict practice scores for pressure ulcer prevention and management (B = −0.01, SE = 0.01, t = −1.05, *p* =.296), with a 95% confidence interval of −0.03 to 0.01. Marital status was also not a significant predictor (B = −0.07, SE = 0.10, t = −0.75, *p* =.457), with a confidence interval of −0.27 to 0.12. Level of education did not significantly predict practice scores (B = −0.01, SE = 0.17, t = −0.06, *p* =.953), with a confidence interval of −0.35 to 0.33. The intercept for this model was 4.29 (SE = 0.42, t = 10.15, *p* <.001), indicating the baseline practices score when age, marital status, and level of education are constant. Knowledge score significantly predicted practice scores (B = 0.58, SE = 0.07, t = 8.51, *p* <.001), with a confidence interval of 0.44 to 0.72, indicating that higher knowledge scores are associated with better practices in pressure ulcer prevention and management, as shown in Table 3.

**Table 3:** Multivariate regression analysis of knowledge, attitudes, and practices scores.

	Parameter	B	SE	t	*p*	95% CI Lower	95% CI Upper
Knowledge Score Regression	Intercept	3.80	0.39	9.82	0.000	3.03	4.57
	Age	0.01	0.01	1.51	0.134	−0.00	0.03
	Marital Status	0.05	0.09	0.57	0.571	−0.13	0.23
	Level of Education	−0.28	0.16	−1.82	0.072	−0.59	0.03
Attitudes Score Regression	Intercept	3.70	0.36	10.32	0.000	2.98	4.41
	Age	0.01	0.01	0.84	0.402	−0.01	0.02
	Marital Status	−0.08	0.08	−0.97	0.333	−0.25	0.09
	Level of Education	0.12	0.14	0.83	0.408	−0.17	0.40
	Intercept	1.20	0.28	4.29	0.000	0.65	1.76
	Knowledge Score	0.67	0.07	9.78	0.000	0.53	0.81
Practices Score Regression	Intercept	4.29	0.42	10.15	0.000	3.45	5.13
	Age	−0.01	0.01	−1.05	0.296	−0.03	0.01
	Marital Status	−0.07	0.10	−0.75	0.457	−0.27	0.12
	Level of Education	−0.01	0.17	−0.06	0.953	−0.35	0.33
	Intercept	1.64	0.27	6.07	0.000	1.11	2.17
	Knowledge Score	0.58	0.07	8.51	0.000	0.44	0.72

## Discussion

The present study aims to evaluate the knowledge, attitudes, and practices of nurses regarding pressure ulcer (PU) prevention and management. The results indicate that most nurses possess a solid understanding of the critical aspects of PU management, a positive attitude towards preventive measures, and commendable practices in their daily patient care routines. These findings are crucial as they highlight the readiness of nurses to combat pressure ulcers, which are a significant concern in healthcare settings due to their impact on patient morbidity and healthcare costs.

The current study reveals that a significant proportion of nurses are knowledgeable about the critical aspects of PU prevention. For instance, most nurses were aware that pressure ulcers could lead to severe illnesses such as septic shock, respiratory failure, and acute renal failure. Additionally, they demonstrated a solid understanding of the Braden Scale for assessing and grading PUs and recognized that skin over bony prominences are more prone to developing pressure ulcers. These findings align with previous studies, suggesting that knowledge about PU prevention is relatively high among nurses^28–30^.

Nuru et al. (2015) conducted an institution-based cross-sectional study at Gondar University Hospital, Northwest Ethiopia, to analyze nurses’ knowledge and practice regarding PU prevention. They found that nurses lacked adequate knowledge and practice in PU prevention, with more experienced nurses and those with higher educational status demonstrating better knowledge and practice^31,32^. Similarly, the study by Kaddourah et al. (2014) in Saudi Arabia found that health professionals had a limited understanding of PU prophylaxis and exhibited negative attitudes towards it. However, increased knowledge was associated with improved attitudes towards PU prevention^33^.

The high mean scores (range 4.02–4.18) on key knowledge items reflect strong agreement among nurses regarding fundamental pressure ulcer prevention concepts, as measured by our 5-point Likert scale. Contrarily, the present study found that nurses generally possessed adequate knowledge about PU prevention, which is a positive development. This disparity could be attributed to differences in educational programs and training sessions provided to nurses in different regions. The high knowledge scores in the current study could be due to effective training programs and continued professional development initiatives^34^.

Our findings reveal that nurses in the studied tertiary care hospital exhibited relatively high knowledge scores (mean Likert scores > 4.0) regarding pressure ulcer (PU) prevention, particularly in areas such as the Braden Scale and risk factors like bony prominences. These results align with a study by Nasreen et al. (2017) in Lahore, Pakistan, where nurses demonstrated moderate to high knowledge levels but identified gaps in practical implementation due to resource constraints^34^. However, our study contrasts with research by Nisa et al. (2024) in the same region, which reported lower adherence to preventive practices, attributed to high patient-to-nurse ratios and limited institutional support^35^.

In broader LMIC contexts, our findings are comparable to studies from Ethiopia and India, where knowledge scores were moderate but attitudes and practices lagged due to systemic challenges. Nuru et al. (2015) and Ebi et al. (2019) highlighted that nurses in Ethiopia had adequate theoretical knowledge but faced barriers like inadequate staffing and equipment, leading to suboptimal practices^31,36^. Similarly, Bhattacharya and Mishra (2015) noted that Indian nurses’ KAP scores were lower than those in our study, emphasizing the role of continuous education in bridging these gaps^37^.

Notably, our participants’ positive attitudes toward preventive measures (e.g., frequent repositioning, wound care) were more pronounced than in studies from Saudi Arabia (Kaddourah et al., 2016) and Sudan (Al-khazali, 2023), where negative attitudes were linked to insufficient training^33,38^. This discrepancy may reflect the impact of localized training programs in our setting, underscoring the importance of context-specific interventions.

The attitudes of nurses towards PU prevention and management were generally positive in the current study. Nurses recognized the importance of frequent repositioning of patients, maintaining wrinkle-free bed sheets, and using appropriate dressings and wound care techniques. These positive attitudes are crucial for the effective implementation of PU prevention strategies^39^.

Miller et al. (2014) investigated critical care nurses’ knowledge of PU prevention and found that while nurses had more understanding about PU staging, there was a knowledge deficit linked to PU prevention practices. This study highlighted the need for continuous education and training to improve nurses’ attitudes and practices regarding PU prevention. The positive attitudes observed in the current study could be a result of such educational interventions, emphasizing the importance of ongoing education in fostering positive attitudes toward PU prevention^40,42,42^.

Barakat-Johnson et al. (2019) explored nurses’ knowledge and attitudes towards pressure injury prevention in Sydney, Australia. They found that nurses were well-versed in pressure injury avoidance and had a positive attitude towards it. Like the current study, Barakat-Johnson et al. found that nurses with more years of experience had better attitudes and practices. This suggests that experience, along with continuous education, plays a vital role in shaping nurses’ attitudes toward PU prevention^43^.

The practices of nurses in PU prevention were positively rated in the current study. Nurses reported adhering to practices such as turning patients every two hours, paying attention to pressure points during skin cleansing, and assessing every patient for the development of PUs upon hospital admission. These practices are essential for reducing the incidence of PUs and ensuring patient safety^44^.

De Meyer et al. (2019) conducted a multicenter study in Belgium to investigate nurses’ and nursing assistants’ knowledge of PU prevention. They found a significant knowledge gap among nurses, indicating the need for improved training and education. The current study, however, found that nurses had significant knowledge and positive attitudes, which translated into good practices in PU prevention. This suggests that the implementation of comprehensive educational programs and continuous professional development can significantly improve nurses’ practices in PU prevention^45^.

The multivariate regression analysis in the current study revealed that age, marital status, and level of education did not significantly predict knowledge, attitudes, or practice scores regarding PU prevention. However, knowledge scores significantly predicted both attitudes and practice scores, indicating that higher knowledge is associated with more positive attitudes and better practices. This finding underscores the importance of enhancing nurses’ knowledge through continuous education and training programs to improve their attitudes and practices toward PU prevention.

Previous studies have also highlighted the importance of knowledge in shaping attitudes and practices. For instance, Kaddourah et al. (2014) found that increased knowledge about PU prevention was associated with improved attitudes among health professionals^33^. Similarly, Barakat-Johnson et al. (2019) and De Meyer et al. (2019) emphasized the role of knowledge in enhancing nurses’ practices in PU prevention^27,43,45^.

The present study’s findings are consistent with several previous studies, highlighting the importance of knowledge, attitudes, and practices in PU prevention. However, there are some discrepancies, particularly regarding the level of knowledge among nurses. While some studies reported a lack of adequate knowledge and practice in PU prevention, the current study found that nurses generally possessed good knowledge and practiced effective preventive measures.

These discrepancies could be attributed to differences in the educational and training programs available to nurses in different regions. The current study’s high knowledge scores could be a result of effective training programs and continuous professional development initiatives provided to nurses in the study setting. This highlights the need for standardized educational programs and training sessions to ensure that all nurses possess adequate knowledge and practice effective PU prevention measures.

The findings of this study highlight several actionable strategies to enhance pressure ulcer (PU) prevention in clinical practice. First, implementing mandatory, quarterly training modules focused on evidence-based guidelines—such as proper use of the Braden Scale, repositioning techniques, and wound care—can address knowledge gaps and standardize practices. Second, introducing structured checklists for PU risk assessment during patient admissions and shift changes can improve adherence to preventive protocols. Third, conducting monthly ward audits with real-time feedback will help identify lapses in practice (e.g., missed repositioning schedules) and reinforce accountability. Finally, ensuring resource allocation, such as providing pressure-relieving mattresses and forming dedicated skin-care teams in high-risk units (e.g., ICU, geriatrics), can mitigate systemic barriers. These interventions align with successful models in LMICs, such as checklist-driven improvements in Ethiopia^31^ and audit-based strategies in Saudi Arabia^33^, demonstrating their feasibility in resource-constrained settings. By adopting these targeted measures, hospitals can bridge the gap between knowledge and practice, ultimately reducing PU incidence.

Besides the contribution to the existing literature, this study has limitations, like the questionnaire, while structured to assess knowledge, attitudes, and practices, was not formally validated or pre-tested in the target population. Future studies should employ validated tools or conduct pilot testing to ensure the instrument’s reliability and cultural appropriateness for the Pakistani nursing context. While the 5-point Likert scale provided clear gradations of agreement, future studies might benefit from including a ‘not applicable’ option for items where nurses lack direct experience.

This study has several limitations. First, the reliance on self-reported data may introduce response bias, as participants might overestimate their knowledge or adherence to best practices. Second, the single-center design limits the generalizability of our findings to other settings, particularly those with differing resource availability or patient demographics. Third, the cross-sectional nature of the study precludes causal inferences about the relationship between knowledge, attitudes, and practices.

Future research should address these limitations through longitudinal assessments to track changes in nurses’ KAP over time, as well as interventional studies (e.g., randomized controlled trials) to evaluate the effectiveness of targeted training programs or checklist implementations. Additionally, multi-center studies across diverse healthcare settings in Pakistan and other LMICs would enhance the external validity of the findings and provide a broader understanding of systemic challenges in PU prevention.

## Conclusion

The present study highlights the importance of knowledge, attitudes, and practices in PU prevention and management among nurses. The findings indicate that nurses generally possess good knowledge, positive attitudes, and commendable practices regarding PU prevention. These findings are consistent with several previous studies, emphasizing the role of continuous education and training in enhancing nurses’ knowledge, attitudes, and practices.

To further improve PU prevention and management, it is essential to implement standardized educational programs and training sessions for nurses. Continuous professional development initiatives should be encouraged to ensure that nurses remain updated with the latest knowledge and practices in PU prevention. By enhancing nurses’ knowledge and fostering positive attitudes, healthcare facilities can significantly reduce the incidence of pressure ulcers and improve patient outcomes.

## Supplementary Information

Below is the link to the electronic supplementary material.

Supplementary Material 1

## Data Availability

The datasets used and/or analysed during the current study available from the corresponding author on reasonable request.

## Abbreviations

PU: Pressure Ulcers
KAP: Knowledge, Attitude, and Practice
SPSS: Statistical Package for the Social Sciences
CI: Confidence Interval
IEC: Institutional Ethical Committee
