# Supplementary material for: Knowledge, attitude, and practice among nurses regarding the prevention of pressure ulcers in a tertiary care hospital: a cross-sectional study
Source: Sci Rep. 2025 Oct 23;15:37019. doi: 10.1038/s41598-025-18303-4 (PMC12549846; doi:10.1038/s41598-025-18303-4)
Supplement: Supplementary file 1 — Supplementary Material 1 [file 41598_2025_18303_MOESM1_ESM.pdf]

Demographic Information:

1. **Age**:
2. **Marital Status**:
   - A. Single
   - B. Married
   - C. Divorced
   - D. Widowed
3. **Level of Education**:
   - A. Diploma in Nursing
   - B. Bachelor of Nursing
   - C. Master of Nursing

Knowledge of Pressure Ulcers:

Multiple-Choice Questions:

1. **Pressure ulcers cause serious illnesses such as septic shock, respiratory failure, and acute renal failure.**
   - A. Strongly Agree
   - B. Agree
   - C. Neutral
   - D. Disagree
   - E. Strongly Disagree
2. **Braden Scale is used for assessing and grading pressure ulcers.**
   - A. Strongly Agree
   - B. Agree
   - C. Neutral
   - D. Disagree
   - E. Strongly Disagree
3. **The skin over bony prominences is more prone to developing pressure ulcers.**
   - A. Strongly Agree

- B. Agree
- C. Neutral
- D. Disagree
- E. Strongly Disagree

4. **Chronic bedridden and wheelchair users are at higher risk of developing pressure ulcers.**
    - A. Strongly Agree
    - B. Agree
    - C. Neutral
    - D. Disagree
    - E. Strongly Disagree
5. **Completely or partially obstructed blood flow to soft tissue leads to the formation of pressure ulcers.**
    - A. Strongly Agree
    - B. Agree
    - C. Neutral
    - D. Disagree
    - E. Strongly Disagree
6. **Neuropathy and paralysis cause pressure ulcers by reducing the sensitivity of the skin.**
    - A. Strongly Agree
    - B. Agree
    - C. Neutral
    - D. Disagree
    - E. Strongly Disagree
7. **The risk of pressure ulcers is increased by skin wetness (e.g., urine incontinence, stool, sweating).**
    - A. Strongly Agree
    - B. Agree

- C. Neutral
- D. Disagree
- E. Strongly Disagree

Attitudes Toward Pressure Ulcer Prevention:

Likert Scale Statements:

1. **Frequent repositioning of patients and wrinkle-free bed sheets prevent pressure ulcers.**
    - A. Strongly Agree
    - B. Agree
    - C. Neutral
    - D. Disagree
    - E. Strongly Disagree
2. **Hydra colloid dressing is used for patients with pressure ulcers.**
    - A. Strongly Agree
    - B. Agree
    - C. Neutral
    - D. Disagree
    - E. Strongly Disagree
3. **Saline water is used to clean the wounds of pressure ulcers.**
    - A. Strongly Agree
    - B. Agree
    - C. Neutral
    - D. Disagree
    - E. Strongly Disagree
4. **Anti-inflammatory drugs delay the healing process of pressure ulcer wounds.**
    - A. Strongly Agree
    - B. Agree
    - C. Neutral

- D. Disagree
- E. Strongly Disagree

5. **Ripple mattresses and cushions have no role in the prevention of pressure ulcers.**
   - A. Strongly Agree
   - B. Agree
   - C. Neutral
   - D. Disagree
   - E. Strongly Disagree
6. **Proper skin care and adequate nutrition prevent pressure ulcers.**
   - A. Strongly Agree
   - B. Agree
   - C. Neutral
   - D. Disagree
   - E. Strongly Disagree
7. **Massage over bony prominences helps in the prevention of pressure ulcers.**
   - A. Strongly Agree
   - B. Agree
   - C. Neutral
   - D. Disagree
   - E. Strongly Disagree

Practices in Pressure Ulcer Prevention:

Multiple-Choice Questions:

1. **How often do you turn patients to prevent bedsores?**
   - A. Every 2 hours
   - B. Every 4 hours
   - C. Once a day
   - D. When the patient complains of discomfort

2. **Do you use pressure-relieving devices regularly?**
    - A. Always
    - B. Often
    - C. Sometimes
    - D. Never
3. **What protocols do you follow for skin assessment during patient care?**
    - A. Assess every patient upon admission
    - B. Assess only at discharge
    - C. No specific protocol
    - D. Assess only high-risk patients
4. **How frequently do you educate patients about pressure ulcer prevention?**
    - A. Regularly
    - B. Occasionally
    - C. Rarely
    - D. Never
5. **Do you adhere to a written turning schedule for patients?**
    - A. Always
    - B. Often
    - C. Sometimes
    - D. Never
6. **How do you position patients to prevent the formation of pressure ulcers?**
    - A. Elevate the head at 30 degrees
    - B. Use pillows under the legs
    - C. Keep the patient flat
    - D. Position is not adjusted
